# High fecal carriage of *bla*_CTX-M_, *bla*_CMY-2_, and plasmid-mediated quinolone resistance genes among healthy Korean people in a metagenomic analysis

**DOI:** 10.1038/s41598-021-84974-4

**Published:** 2021-03-12

**Authors:** Jieun Kim, Kye-Yeung Park, Hoon-Ki Park, Hwan-Sik Hwang, Mi-Ran Seo, Bongyoung Kim, Youna Cho, Mina Rho, Hyunjoo Pai

**Affiliations:** 1 Division of Infectious Disease, Department of Internal Medicine, College of Medicine, Hanyang University, 222 Wangsimni-ro, Seongdong-gu, Seoul, 04763 Republic of Korea; 2 Department of Family Medicine, College of Medicine, Hanyang University, Seoul, 04763 Republic of Korea; 3 Advanced BioVision Inc, #129, Gaetbeol-Ro, Yeonsu-Gu, Incheon, 21999 Republic of Korea; 4 Department of Computer Science and Engineering, Hanyang University, Seoul, 04763 Republic of Korea; 5 Department of Biomedical Informatics, Hanyang University, Seoul, 04763 Republic of Korea

**Keywords:** Antimicrobials, Metagenomics

## Abstract

To characterize the carriage of antibiotic resistance genes (ARGs) in the gut microbiome of healthy individuals. Fecal carriage of ARGs was investigated in 61 healthy individuals aged 30 to 59 years through whole metagenome sequencing of the gut microbiome and a targeted metagenomic approach. The number of ARGs in the gut microbiome was counted and normalized per million predicted genes (GPM). In the Korean population, the resistome ranged from 49.7 to 292.5 GPM (median 89.7). Based on the abundance of ARGs, the subjects were categorised into high (> 120 GPM), middle (60‒120 GPM), and low (< 60 GPM) ARG groups. Individuals in the high ARG group tended to visit hospitals more often (*P* = 0.065), particularly for upper respiratory tract infections (*P* = 0.066), and carried more *bla*_CTX-M_ (*P* = 0.008). The targeted metagenome approach for *bla* and plasmid-mediated quinolone resistance (PMQR) genes revealed a high fecal carriage rate; 23% or 13.1% of the subjects carried *bla*_CTX-M_ or *bla*_CMY-2_, respectively. Regarding PMQR genes, 59% of the subjects carried PMQR, and 83% of them harboured 2‒4 PMQR genes (*qnrB* 44.3%, *qnrS* 47.5% etc.). The presence of *bla*_CTX-M_ correlated with ARG abundance in the gut resistome, whereas PMQR genes were irrelevant to other ARGs (*P* = 0.176). Fecal carriage of *bla*_CTX-M_ and PMQR genes was broad and multiplexed among healthy individuals.

## Introduction

Antimicrobial resistance (AMR) is currently one of the most important issues in public health. Therefore, overcoming AMR is a major challenge for public health on a global scale. Antibiotic resistance genes (ARGs) are ancient^1^, but recent antibiotic usage in humans and livestock effectively imposes a selection pressure on ARGs. The digestive tract of humans and animals is the main reservoir where ARG exchange occurs, and antibiotic treatment select for the overgrowth of resistant bacteria^2^.

The World Health Organization defined extended-spectrum cephalosporins and fluoroquinolones (FQs) as one of the highest priority critically important antimicrobials^3^. However, in the last 10 to 20 years, the prevalence of extended-spectrum β-lactamase (ESBL) and plasmid-mediated quinolone resistance (PMQR) genes has increased dramatically worldwide^4–6^. Many studies have reported the fecal carriage rate of ESBLs (especially CTX-M) among healthy individuals across many countries^5^, and demonstrated high levels of fecal ESBL carriage in Southeast Asian, Eastern Mediterranean, and Western Pacific countries. Since the first identification of PMQR genes^7^, their prevalence has increased worldwide^6,8^. The close association between PMQR and *bla*_CTX-M_ alleles has raised further concerns^4,8^. Several studies have revealed that travellers returning from East Asian countries temporarily harboured *bla*_CTX-M_ or PMQR genes^9,10^.

The objective of this study was to evaluate the fecal carriage of ARGs in healthy Korean individuals. First, we analyzed the diversity and abundance of ARGs in the gut microbiome using whole metagenome sequencing. Second, we used a targeted metagenomic approach based on polymerase chain reaction (PCR) and sequencing specific for *bla*_CTX-M,_ plasmid-AmpC β-lactamase (pAmpC), and PMQR genes. Lastly, we characterised the association between the fecal carriage of *bla*_CTX-M_, p-AmpC, and PMQR genes and the abundance of non-redundant ARGs in the gut microbiome and daily lifestyle of the enrolled subjects.

## Methods

### Study design

This study was conducted from June to October 2017 on a group of healthy individuals who visited the Hanyang University Health Promotion Centre for health screening services in Seoul. Participants who had zero scores based on the Charlson comorbidity index^11^ and no admission history within the last year were defined as healthy. Sixty-one subjects aged 30 to 59 years were enrolled. After obtaining written informed consent, the patients were requested to answer a lifestyle questionnaire and feces were collected from individual participants. The study protocol was approved by the institutional review boards (Hanyang University Hospital institutional review boards 2017–06-001). All methods were carried out in accordance with relevant guidelines and regulations.

### Personal information

The questionnaire included the following information: age, sex, water consumed, medications, diet habit and the presence of companion animals. Medical history and the frequency and reasons for visiting a hospital within the last year were also investigated.

### Fecal DNA preparation

Each sample was thoroughly mixed using a spatula and divided into 250‒300 mg aliquots. Total DNA was extracted using a Fast DNA SPIN Kit for Faeces (MP Biomedicals, #116,570,200), following the manufacturer’s instructions.

### Analysis of metagenome sequencing data

Metagenome sequencing data (n = 61) of the gut microbiome from our study were obtained from the European Nucleotide Archive (ENA; accession number PRJEB33013). For every sample, the length of the paired-end reads was 151 bp with an insert size of 350 bp. A three-step procedure was performed. First, filtered reads were assembled into contigs using MEGAHIT^12^ with default options. Genes were predicted from the contigs (> 500 bp) using FragGeneScan^13^ with the options of no sequencing error (-w 0 -t complete). Lastly, ARGs were identified using the CARD databaseS1^14^ and BLASTp^15^ with an e-value threshold of 1 × 10^–10^, similarity > 70%, and reference coverage > 70%. The resistance genes were classified into 20 ARG classes (Supplementary Table ). The abundance of non-redundant ARGs was measured using the number of ARGs per million genes (GPM) in each sample as follows:

$${\text{GPM}} = \frac{{{\text{Number}}\;{\text{of}}\;{\text{ARGs}}\;{\text{identified}} \times 10^{6} }}{{{\text{Number}}\;{\text{of}}\;{\text{genes}}\;{\text{predicted}}}}$$

### Detection of ARGs by polymerase chain reaction and sequencing

The following AMR genes: *bla*_CTX-M_ (*bla*_CTX-M-1_ and *bla*_CTX-M-9_ group), p-AmpC (*bla*_CMY-1_, *bla*_CMY-2_, *bla*_DHA_, and *bla*_FOX_), carbapenemases (*bla*_IMP_, *bla*_NDM_, *bla*_KPC_, and *bla*_VIM_), and PMQR (*qnrA*, *qnrB*, *qnrD*, *qnrS*, *qepA*, and *aac(6′)-Ib-cr*) were detected using PCR with specific primers and conditions summarised in Supplementary Table S2^16–25^. After electrophoresis on 2% agarose gel, the resistance alleles were identified by sequencing the PCR products and comparing the sequences with those in the GenBank database.

### Statistical analysis

To compare demographic characteristics and health information, SPSS version 24.0 for Windows (SPSS Inc., Armonk, NY, USA) was used. Pearson’s Chi-square test or Fisher’s exact test was used to analyze categorical variables, and the independent *t*-test or Mann‒Whitney U-test was used to analyze continuous variables. Spearman’s rank correlation test was performed to evaluate the relationship between the two variables. A *P*-value of < 0.05 in a two-tailed test was considered significant.

### Access to data

Metagenome sequencing data of the gut microbiome were obtained from the European Nucleotide Archive (ENA; accession number PRJEB33013).

## Results

### Demographic data and personal information of 61 healthy Korean individuals

A total of 61 healthy individuals (61 fecal samples) were included in this study. The median age was 46 years (1Q, 3Q; 37, 51) and 47.5% of the subjects were female. Proportion of gender was similar among the age of 30 s, 40 s and 50 s. The median body mass index was 23.3 (1Q, 3Q; 21.6, 25). Personal histories including water, probiotic, and vitamin intake as well as the presence of a companion animal are shown in Table 1. As for the medical history, 11.5% of the subjects reported that they had been prescribed antibiotics within the past year, 34.4% did not visit the hospital, and 50.8% visited the hospital less than three times within the past year. For the treatment of upper respiratory tract infections (URIs), 47.5% of the participants did not visit the hospital and 49.2% reported visiting the hospital fewer than three times, and 3.3% visited four to six times; for dental problems, 44.2% visited a dental clinic.

**Table 1:** Comparison of demographic data and personal or medical history between people with HARG and LARG in the gut.

Characteristics	N (%)	Total (N = 61)	HARG (N = 10)	LARG (N = 10)	*P* value
Gender	Female	29 (47.5)	5 (50)	4 (40)	1
	Male	32 (52.5)	5 (50)	6 (60)	
Age	Median (1Q, 3Q)	46 (39, 51)	43 (35.5, 54)	46.5 (42.5, 49.3)	0.622
Age & gender	30 s	17 (27.9)	4 (40)	1 (10)	0.768*
	Female	9 (52.9)	3 (75)	0 (0)	0.4
	40 s	26 (42.6)	2 (20)	7 (70)	
	Female	11 (42.3)	0 (0)	3 (42.9)	0.417
	50 s	18 (29.5)	4 (20)	2 (20)	
	Female	9 (50)	2 (50)	1 (50)	1
Companion animal	None	54 (88.5)	10 (100)	10 (100)	
Water	Bottled Water	36 (59)	5 (50)	8 (80)	0.35
	Purified Water	30 (49.2)	7 (70)	4 (40)	0.37
	Tap water	11 (18)	1 (10)	0	1
Medication	Probiotics	12 (19.7)	5 (50)	2 (20)	0.35
	multi-vitamin	18 (29.5)	4 (40)	2 (20)	0.628
Stool habitus	Regular	46 (75.4)	8 (80)	6 (60)	0.628
**Medical history within 1 year**					
Antibiotic use	none	54 (88.5)	9 (90)	8 (80)	1
Number of hospital visits	> 6	2 (3.3)	1 (10)	0	0.066*
	4–6	7 (11.5)	1 (10)	1 (10)	
	≤ 3	31 (50.8)	6 (60)	2 (20)	
	none	21 (34.4)	2 (20)	7 (70)	
Hospital visit d/t febrile condition	≤ 3	5 (8.2)	1 (10)	0	1
	none	56 (91.8)	9 (90)	10 (100)	
Hospital visit d/t upper respiratory infection	4–6	2 (3.3)	0	1 (10)	0.065*
	≤ 3	30 (49.2)	7 (70)	0	
	none	29 (47.5)	3 (30)	9 (90)	
Hospital visit d/t dental problem	4–6	3 (4.9)	0	0	0.65*
	≤ 3	24 (39.3)	5 (50)	3 (30)	
	none	34 (55.7)	5 (50)	7 (70)	

*p for trend.

*HARG* high antibiotic resistance gene, *LARG* low antibiotic resistance gene, *d/t* due to, *Q* quartile.

### Different phenotypes between the groups with high and low ARG abundance

**Figure 1:**
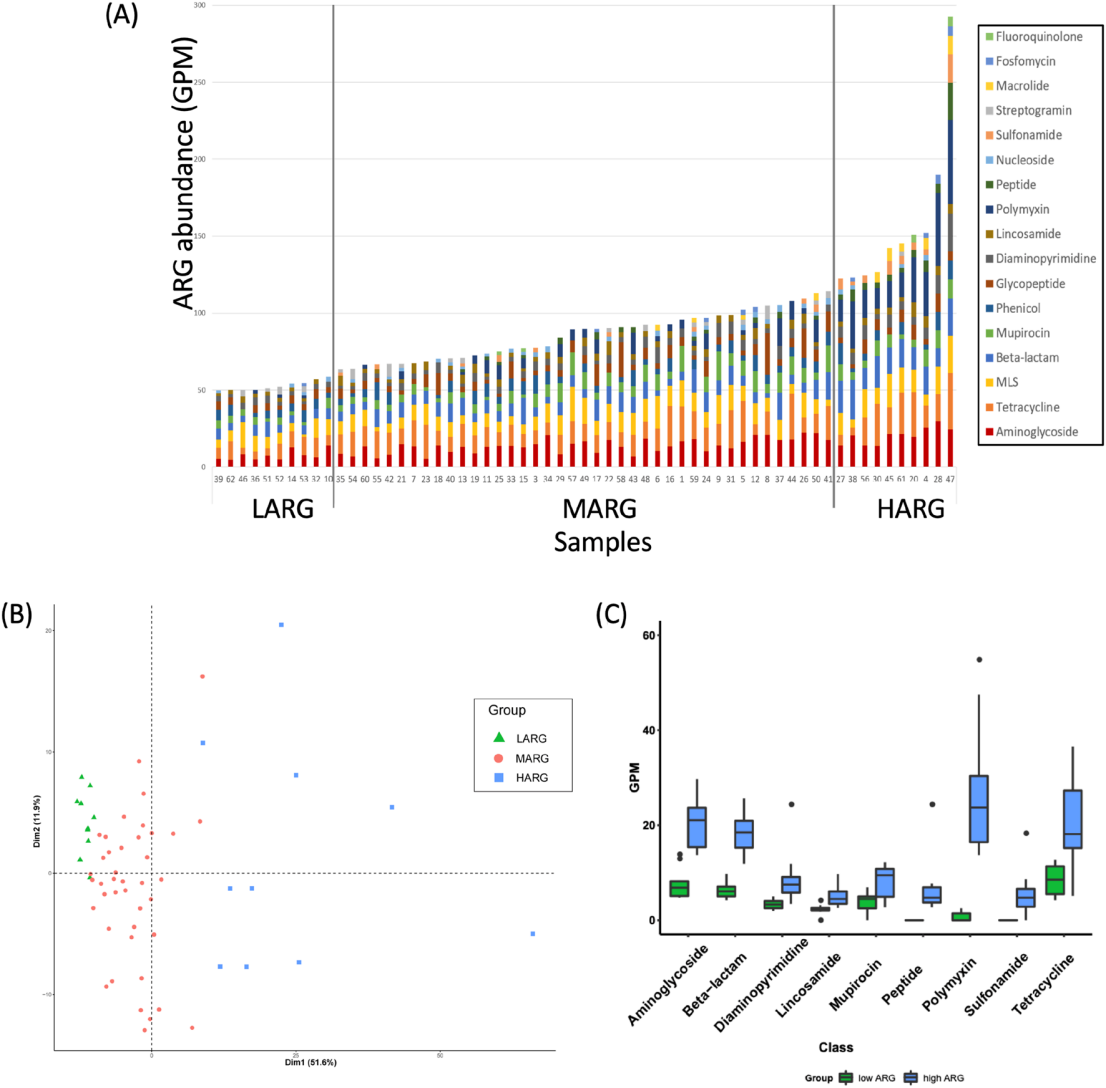
Distribution of antibiotic resistance determinants in the guts of healthy Korean subjects. (**A**) The distribution of resistance determinants in LARG, MARG, and HARG groups. Sample numbers are shown in x-axis. HARG consisted of 10 subjects who had a higher abundance (> 120 GPM) of antibiotic resistance genes. LARG consisted of 10 subjects that had lower abundance (< 60 GPM) of antibiotic resistance genes. MARG was between LARG and HARG, which had ARGs of higher than 60 GPM and lower than 120 GPM (**B**) Principal component analysis of resistance determinants in HARG, MARG and LARG. (**C**) Nine antibiotic determinants were significantly enhanced in HARG of the two groups (*P*-value < 0.01). *HARG* high antibiotic resistance genes group, *MARG* middle antibiotic resistance genes group, *LARG* low antibiotic resistance genes group.

Figure 1A presents the abundance of non-redundant ARGs in the gut microbiome of healthy Korean individuals. In the Korean population, the resistome ranged from 49.7 to 292.5 GPM, with a median value of 89.7. Aminoglycoside was the most abundant antibiotics resistance determinant out of 20 (supplementary Table S1), which was followed by tetracycline, macrolides-lincosamides-streptogramins shared (MLS), and beta-lactam (Fig. 1A, supplementary Table S3). The prevalence of these determinants was more than 98% of the samples.

Based on the abundance of non-redundant ARGs in the gut, we categorised the 61 healthy individuals into the following three groups: high ARG (HARG), consisting of 10 subjects with ARGs > 120 GPM; low ARG (LARG), consisting of 10 subjects with ARGs < 60 GPM; and middle ARG (MARG), consisting of 41 subjects with ARGs between HARG and LARG (Fig. 1A). The median values for the abundance of ARG in HARG, MARG, and LARG (range) were 142.5 (124.1, 160.7), 89.7 (70.7, 97.7), and 51.8 GPM (50.0, 55.1), respectively. Principal component analysis showed that HARG is completely separate from LARG (Fig. 1B). In terms of resistance abundance, nine resistance determinants were significantly more abundant in HARG than that in LARG group. Particularly, β-lactam was the most significant, followed by aminoglycoside, diaminopyrimidine, lincosamide, mupirocin, peptide, polymyxin, sulphonamide, and tetracycline (*p*-value < 0.01; Fig. 1C).

### Comparison of the lifestyles of high, middle, and low ARG abundance groups

On comparing the lifestyle of the three groups, no difference was observed in water and medication intake and the presence of a companion animal. However, the medical history within the last year showed a difference; the number of hospital visits and hospital visit due to URI correlated with ARG abundance with a marginal significance (*p* for trend = 0.066, *p* for trend = 0.065, respectively; Table 1). In terms of the diet habit and fecal carriage of ARG, we could not find a significant difference in diet habit among people with HARG, MARG and LARG (Supplementary Table S4).

### Fecal carriage rate of bla_CTX-M_, p-AmpC, and PMQR genes

**Figure 2:**
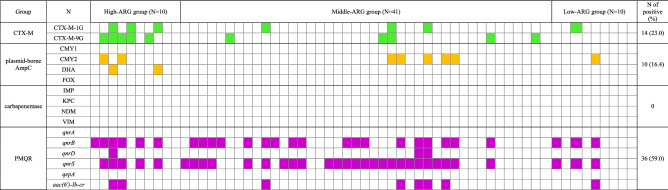
Faecal carriage of alleles of CTX-M, plasmid-mediated AmpC (p-AmpC) plasmid-mediated carbapenemases, and plasmid-mediated quinolone resistance (PMQR) stratified based on the three groups of 61 healthy subjects confirmed by polymerase chain reaction (PCR) and sequencing for each specific allele. HARG, high ARG group; MARG, middle ARG group; LARG, low ARG group.

To investigate the fecal carriage of *bla*_*CTX-M*_, p-AmpC, and PMQR genes more precisely, we performed PCR and sequencing for each allele using the fecal samples of 61 individuals. Overall, 18 of the 61 subjects (29.5%) carried *bla*_CTX-M_, p-AmpC, or both alleles (Table 2, Fig. 2). The fecal carriage rate of *bla*_CTX-M_ was 23% (14/61); that of CTX-M-1 group and CTX-M-9 group was 11.5% (7/61) and 16.4% (10/61), respectively and three subjects (5%) carried alleles of both groups. With respect to p-AmpC alleles, 10 of the 61 subjects (16.4%) harboured the *bla*_CMY-2_ or *bla*_DHA_ group in their gut; 13.1% (8/61) for *bla*_CMY-2_ and 3% (2/61) for *bla*_DHA_, but none of the subjects carried *bla*_CMY-1_ or *bla*_FOX_. None of the 61 healthy subjects carried plasmid-mediated carbapenemase alleles in their gut.

**Table 2:** Faecal carriage rate of CTXM, plasmid-mediated AmpC, and plasmid-mediated quinolone resistance alleles in 61 healthy people.

Antibiotic resistance alleles	% (number)				*P* for trend
	Total	HARG	MARG	LARG	
CTX-M	23% (14)	60% (6)	17.1% (7)	10% (1)	0.008
CTX-M-1G	11.5% (7)	30% (3)	7.3% (3)	10% (1)	0.164
CTX-M-9G	16.4% (10)	50% (5)	12.2% (5)	0% (0)	0.003
Plasmid-mediatedAmpC	16.4% (10)	40% (4)	12.2% (5)	10% (1)	0.072
CMY-1	0% (0)	0% (0)	0% (0)	0% (0)	
CMY-2	13.1% (8)	20% (2)	12.2% (5)	10% (1)	0.511
FOX	0% (0)	0% (0)	0% (0)	0% (0)	
DHA	3.3% (2)	20% (2)	0% (0)	0% (0)	0.013
Plasmid-mediatedquinolone resistance	59% (36)	60% (6)	65.9% (27)	30% (3)	0.176
*qnrA*	0% (0)	0% (0)	0% (0)	0% (0)	
*qnrB*	44.3% (27)	60% (6)	43.9% (18)	30% (3)	0.18
*qnrD*	4.9% (3)	10% (1)	4.9% (2)	0% (0)	0.213
*qnrS*	54.1% (33)	50% (5)	61% (25)	30% (3)	0.373
*qepA*	0% (0)	0% (0)	0% (0)	0% (0)	
*aac(6′)-Ib-cr*	13.1% (8)	10% (2)	12.2% (5)	10% (1)	0.511
Total (median, [1Q, 3Q])		2 (0.8, 4.3)	1 (0, 2)	0 (0, 0.8)	0.035*

Plasmid-mediated carbapenemase alleles were not identified.

*HARG* high antibiotic resistance genes group, *MARG* middle antibiotic resistance genes group, *LARG* low antibiotic resistance genes group.

CTX-M-1G, CTX-M-1 group; CTX-M-9G, CTX-M-9 group.

*By Kruskal‒Wallis test.

With respect to PMQR genes, 36 healthy individuals carried PMQR in their gut in general (59%); *qnrA* 0%, *qnrB* 44.3% (27/61), *qnrD* 4.9% (3/61), *qnrS* 47.5% (29/61), and *aac(6′)-Ib-cr* 13.1% (8/61), respectively (Table 2). Overall, 30 out of the 36 subjects (83%) harboured two to four PMQR genes; three subjects (4.9%) harboured *qnrB, qnrD, qnrS*, and *aac(6′)-Ib-cr*: 7 (11.5%), *qnrB, qnrS*, and *aac(6′)-Ib-cr*: 20 (32.8%), *qnrB* and *qnrS*: one (2.4%) and nine (14.8%) *qnrB* or *qnrS* alone, respectively (Fig. 2).

### Comparison of fecal ARG carriage rate among HARG, MARG, and LARG groups

The fecal carriage rate of CTX-M, p-AmpC, and PMQR genes was compared among HARG, MARG, and LARG groups (Table 2). The HARG group carried more *bla*_CTX-M_ than MARG or LARG (60% [6/10], 17% [7/41], and 10% [1/10], respectively, *p* for trend = 0.008). The fecal carriage rate of p-AmpC alleles (*bla*_CMY-2_ and *bla*_DHA_ groups) was 40% (4/10), 12.2% (5/41), and 10% (1/10), in HARG, MARG, LARG groups, respectively (*p* for trend = 0.072).

Unlike CTX-M alleles, the fecal carriage rate of total PMQR genes was not different among HARG, MARG, and LARG groups (60% [6/10], 65.9% [27/41], and 30% [3/10], respectively; *p* for trend = 0.176). Individual *qnr* genes, such as *qnrB, qnrD, qnrS*, and *aac(6′)-Ib-cr*, did not show a difference in prevalence among HARG, MARG, and LARG groups (*p* for trend = 0.18, 0.213, 0.373, and 0.511, respectively).

The median number of carriage for CTX-M, p-AmpC, and PMQR genes was two in HARG, one in MARG, and zero in LARG groups (*P* = 0.035 by Kruska‒Wallis test; Table 2).

### Associated presence of CTX-M, p-AmpC, and PMQR genes and their relationships with medical history or the relative abundance of ARG groups in the gut resistome

**Figure 3:**
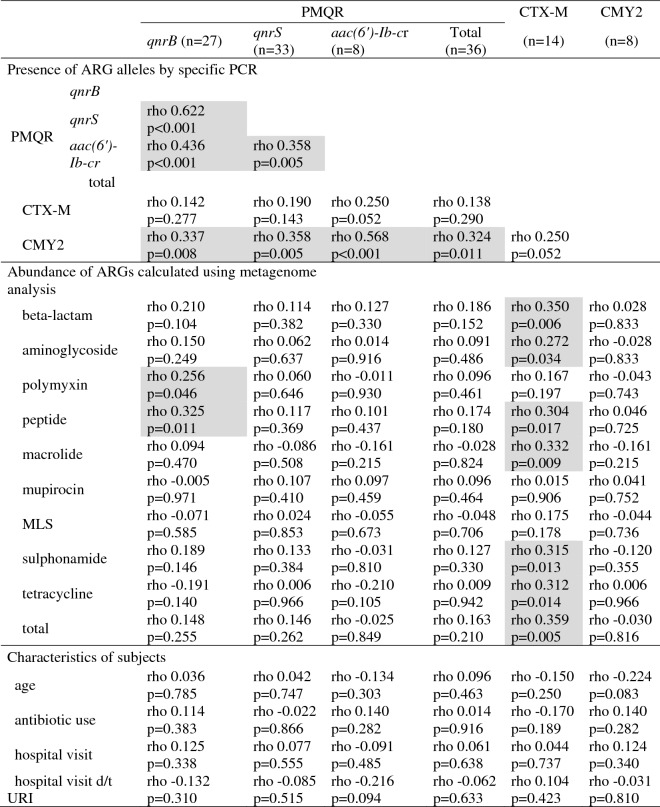
Associated presence of CTX-M, plasmid-mediated AmpC, and PMQR genes and their relationship with the abundance of ARGs and medical history. The shaded box represents a statistically significant relationship between two variables. *PMQR* plasmid-mediated quinolone resistance alleles, *MLS* macrolides-lincosamides-streptogramins shared, *URI* upper respiratory infection.

Associated presence among each ARG allele was analyzed and is presented in Fig. 3. The presence of individual *qnrB*, *qnrS*, and *aac(6′)-Ib-cr* was linked to one another, and carriage of the *bla*_CMY-2_ group was significantly associated with the carriage of most PMQRs as well (*qnrB, qnrS,* and *aac(6′)-Ib-cr*). However, the presence of CTX-M alleles was not associated with that of other resistance alleles. The presence of CTX-M, CMY2, and PMQR genes in each individual was also analyzed using the abundance of ARG groups classified based on antibiotic resistance ontology^14^, which was determined using metagenome analysis of their gut microbiomes (Fig. 3). Carriage of CTX-M alleles showed a significant association with the abundance of most ARG groups, β-lactam, aminoglycoside, peptide, macrolide, sulphonamide, tetracycline, and total ARG. However, CMY2 and PMQR genes were not related with the abundance of ARG groups in the gut resistome except *qnrB* vs. polymyxin and peptide.

Carriage of those resistance alleles and demographics or medical history showed no significant correlation (Fig. 3).

## Discussion

This study found that healthy Korean individuals carried high frequencies of *bla*_CTX-M_, *bla*_CMY-2_, and PMQR genes in their gut. The fecal carriage of ARGs in healthy individuals was analyzed by metagenome sequencing of faeces and ARG-specific PCR, along with lifestyle questionnaires.

Among healthy people, the abundance of ARGs showed great variance. We had expected that diet habit and medical history influenced the ARG abundance in gut. Unfortunately, we could not verify the association between diet habit and ARG abundance, but frequency of clinic visit was marginally associated with ARG abundance in the gut. Two subjects of sample 47 and 28 showed an exceptionally high abundance of ARGs in the gut (Fig. 1A), and all the ARG determinants increased. Interestingly, abundance of *Escherichia* genus increased over 100-fold more than median value of 61 people in bacterial composition (data not shown), which suggests a recent antibiotic usage in both subjects, despite individuals not mentioning antibiotic usage in life style survey.

Interestingly, the fecal carriage of *bla*_CTX-M_ and PMQR genes in healthy individuals looked different in distribution; the presence of *bla*_CTX-M_ was proportional to the abundance of ARGs in the gut microbiome and its presence correlated with many ARG groups, such as β-lactam, aminoglycoside, macrolide, sulphonamide, and total ARGs in the gut microbiome. However, PMQR genes were observed regardless of ARG abundance in the gut, except *qnrB* vs. polymyxin and peptide alleles. Considering that subjects in the HARG group tended to visit clinics more often, especially due to URI, this may imply that *bla*_CTX-M_ carriage is associated with the prescribed antibiotic intake.

With respect to PMQR genes, the fecal carriage rate was very high in healthy Korean individuals regardless of the relative ARG abundance in the gut microbiome. Many individuals carried multiple PMQR genes at the same time; 5% of the subjects harboured four PMQR genes and 11.5% harboured three PMQR genes, indicating frequent exposure to PMQR during daily life. In the metagenome analysis of the gut microbiome, *qnr* alleles were not frequently encountered; we found *qnr* genes in only 3 out of 61 individuals in this study (data not shown), which indicates a high prevalence but low density of PMQR genes in healthy Korean individuals.

Many previous studies have presented an association between PMQR and *bla*_CTX-M_ alleles^5,6,26^; however, an association between these alleles was not identified in this study. Instead, an association between *bla*_CMY-2_ and PMQR genes was observed. A recent study published in 2018 demonstrated a high prevalence of *bla*_CMY-2_ in blood isolates of *Escherichia coli* from Singapore, suggesting a high prevalence of *bla*_CMY-2_ in Asian countries^27^. CMY β-lactamase is predominant p-AmpC in the animal sector—especially broiler and meat—along with *qnr*s^6,28^. These findings suggest a common environmental source—such as broiler meat, contaminated water, or vegetables—for exposure to *bla*_CMY-2_ and PMQR genes.

In this study, the fecal carriage rate of *bla*_CTX-M_ and/or *bla*_CMY-2_ was 29.5%, and that of PMQR genes was 59% in healthy Korean individuals. In our metagenomic analyses of the gut microbiome, *bla*_CTX-M_ and *bla*_CMY-2_ contigs were observed in 4 out of the 61 individuals (6.6%) and 1 out of the 61 individuals (1.6%), respectively^29^, indicating a relatively low density of *bla*_CTX-M_ and *bla*_CMY-2_ in the guts of healthy individuals without antibiotic intake.

Interestingly, a recent study on antibiotic resistance in *E. coli* isolated from community onset acute pyelonephritis patients in Korea—in 2017 and 2018—showed that resistance rates against FQ and cefotaxime were 33.5% and 34.8%, respectively^30^. The fecal carriage rate of ARGs in this study represents the resistance rates of *E. coli* in community infections.

The advantage of this study is that the state of ARG carriage was investigated in detail along with the gut resistome and lifestyle. Despite this strength, some limitations were also there; first, the number of enrolled subjects was not sufficient to represent healthy Korean individuals. Second, we could not directly show the risk factors in lifestyle or medical history for the fecal carriage of antibiotic resistance alleles.

## Conclusion

In conclusion, *bla*_CTX-M_, *bla*_CMY-2_, and PMQR genes were broadly distributed in the gut microbiome of healthy Korean individuals. The fecal carriage rate of *bla*_CTX-M_ was 23%, mostly in individuals with a high abundance of gut resistome. Conversely, 59% of the healthy individuals carried multiple PMQR genes in their gut microbiome regardless of ARG abundance in their guts, which could indicate frequent exposure to PMQR genes.

## Supplementary Information

Supplementary Information
