# Supplementary material for: High fecal carriage of blaCTX-M, blaCMY-2, and plasmid-mediated quinolone resistance genes among healthy Korean people in a metagenomic analysis
Source: Sci Rep. 2021 Mar 12;11:5874. doi: 10.1038/s41598-021-84974-4 (PMC7955109; doi:10.1038/s41598-021-84974-4)
Supplement: Supplementary file 1 — Supplementary Information [file 41598_2021_84974_MOESM1_ESM.docx]

Title: High fecal carriage of blaCTX-M, blaCMY-2, and plasmid-mediated quinolone resistance genes among healthy Korean people in a metagenomic analysis

Jieun Kim

Department of Internal Medicine, College of Medicine, Hanyang University, Seoul, 04763, Republic of Korea

Kye-Yeung Park

Department of Family Medicine, College of Medicine, Hanyang University, Seoul, 04763, Republic of Korea

Hoon-Ki Park

Department of Family Medicine, College of Medicine, Hanyang University, Seoul, 04763, Republic of Korea

Hwan-Sik Hwang

Department of Family Medicine, College of Medicine, Hanyang University, Seoul, 04763, Republic of Korea

Mi-Ran Seo

Advanced BioVision Inc., #129, Gaetbeol-Ro, Yeonsu-Gu, Incheon, 21999, Republic of Korea

Bongyoung Kim

Department of Internal Medicine, College of Medicine, Hanyang University, Seoul, 04763, Republic of Korea

Youna Cho

Department of Computer Science and Engineering, Hanyang University, Seoul, 04763, Republic of Korea

Mina Rho*

Department of Computer Science and Engineering, Hanyang University

Department of Biomedical Informatics, Hanyang University, Seoul, 04763, Republic of Korea

Hyunjoo Pai*

Department of Internal Medicine, College of Medicine, Hanyang University, Seoul, 04763, Republic of Korea

*Equally contributed as the corresponding authors

Corresponding author

Hyunjoo Pai*

Division of Infectious disease

Department of Internal Medicine

Hanyang University College of Medicine

222 Wangsimni-ro, Seongdong-gu, Seoul, 04763, Korea

Tel: 82-2-2290-8356, Fax: 82-2-2298-9183

E-mail address: [paihyunjoo@gmail.com](mailto:paihyunjoo@gmail.com)

Supplementary Table S1. Summary of determinants and the associated genes

| Determinant | Class | Gene  Counts |
| --- | --- | --- |
| Aminocoumarin | antibiotic resistant DNA topoisomerase subunit | 1 |
| Aminoglycoside | aminoglycoside acetyltransferase (AAC) | 81 |
|  | aminoglycoside phosphotransferase (APH) | 34 |
|  | aminoglycoside nucleotidyltransferase (ANT) | 36 |
|  | 16S ribosomal RNA methyltransferase | 11 |
| Beta-lactam | class A beta-lactamase | 660 |
|  | class C beta-lactamase | 287 |
|  | class B (metallo-) beta-lactamase | 162 |
|  | class D beta-lactamase | 292 |
|  | general bacterial porin with reduced permeability to beta-lactams | 2 |
|  | beta-lactam resistant penicillin-binding proteins | 6 |
| Diaminopyrimidine | trimethoprim resistant dihydrofolate reductase dfr | 28 |
| Fluoroquinolone | quinolone resistance protein (qnr) | 95 |
| Fosfomycin | murA transferase | 1 |
|  | Fom phosphotransferase family | 2 |
|  | fosfomycin inactivation enzyme | 11 |
|  | fosC phosphotransferase family | 2 |
| Fusidic acid | fusidic acid inactivation enzyme | 4 |
| Glycopeptide | Bleomycin resistant protein | 1 |
|  | gene(s) or protein(s) associated with glycopeptide resistance cluster | 51 |
| LMS | Cfr 23S ribosomal RNA methyltransferase | 6 |
|  | Erm 23S ribosomal RNA methyltransferase | 33 |
| Lincosamide | lincosamide nucleotidyltransferase (LNU) | 8 |
|  | LlmA 23S ribosomal RNA methyltransferase | 1 |
| Macrolide | macrolide inactivation enzyme | 16 |
|  | non-erm 23S ribosomal RNA methyltransferase (G748) | 4 |
|  | gimA family macrolide glycosyltransferase | 1 |
| Mupirocin | ileS | 3 |
| Nucleoside | streptothricin acetyltransferase (SAT) | 3 |
|  | tunicamycin resistance protein | 1 |
| Peptide | undecaprenyl pyrophosphate related proteins | 2 |
|  | defensin resistant mprF | 5 |
|  | Bah amidohydrolase | 1 |
|  | edeine acetyltransferase | 1 |
|  | non-erm 23S ribosomal RNA methyltransferase (A1067) | 1 |
|  | viomycin phosphotransferase | 1 |
| Phenicol | chloramphenicol acetyltransferase (CAT) | 35 |
|  | chloramphenicol phosphotransferase | 1 |
| Polymyxin | phosphoethanolamine transferase conferring colistin resistance | 12 |
|  | lipid A phosphatase | 1 |
| Rifamycin | rifampin inactivation enzyme | 11 |
|  | rifamycin-resistant beta-subunit of RNA polymerase (rpoB) | 1 |
|  | rifampin-resistant RNA polymerase-binding protein | 1 |
| Streptogramin | streptogramin inactivation enzyme | 11 |
| Sulphonamide | sulphonamide resistant dihydropteroate synthase | 3 |
| Tetracycline | tetracycline-resistant ribosomal protection protein | 11 |
|  | tetracycline inactivation enzyme | 2 |

Supplementary Table S2. Primers and conditions for PCR of carbapenemase, CTX-M, plasmid-mediated AmpC, and plasmid-mediated quinolone resistance genes

| Targeted gene | Primer sequences (5' →3') | Amplicon size (bp) | Ref* |
| --- | --- | --- | --- |
| IMP | F: GGAATAGAGTGGCTTAAYTCTC | 188 | 15 |
|  | R: CCAAACYACTASGTTATCT |  |  |
| NDM | F: GGTTTGGCGATCTGGTTTTC | 621 | 16 |
|  | R: CGGAATGGCTCATCACAGTC |  |  |
| KPC | F: TGTTGCTGAAGGAGTTGGGC | 340 | 17 |
|  | R: ACGACGGCATAGTCATTTGC |  |  |
| VIM | F: CGCGGAGATTGARAAGCAAA | 247 | 17 |
|  | R: CGCAGCACCRGGATAGAARA |  |  |
| CTXM-1 group | F: AAAAATCACTGCGCCAGTTC | 415 | 18 |
|  | R: AGCTTATTCATCGCCACGTT |  |  |
| CTXM-9 group | F: CAAAGAGAGTGCAACGGATG | 205 | 18 |
|  | R: ATTGGAAAGCGTTCATCACC |  |  |
| CMY1 | F: GCTGCTCAAGGAGCACAGGATCCCG | 522 | 19 |
|  | R: GGCACATTGACATAGGTGTGGTGCATG |  |  |
| CMY2 | F: ACTGGCCAGAACTGACAGGCAAA | 466 | 19 |
|  | R: GTTTTCTCCTGAACGTGGCTGGC |  |  |
| DHA | F: CTTTCACAGGTGTGCTGGGTGCG | 403 | 19 |
|  | R: CCGTACGCATACTGGCTTTGCGC |  |  |
| FOX | F: CATGGGGTATCAGGGAGATGCC | 218 | 19 |
|  | R: GCCGCTGCTCGCCCATCG |  |  |
| *qnrA* group | F: ATTTCTCACGCCAGGATTTG | 516 | 20 |
|  | R: GATCGGCAAAGGTTAGGTCA |  |  |
| *qnrB* group | F: GATCGTGAAAGCCAGAAAGG | 476 | 20 |
|  | R: ATGAGCAACGATGCCTGGTA |  |  |
| *qnrD* group | F: AACAAGCTGAAGCGCCTG | 580 | 21 |
|  | R: CGAGATCAATTTACGGGGAATA |  |  |
| *qnrS* group | F: ACTGCAAGTTCATTGAACAG | 431 | 22 |
|  | R: GATCTAAACCGTCGAGTTCG |  |  |
| *qepA* group | F: GCAGGTCCAGCAGCGGGTAG | 218 | 23 |
|  | R: CTTCCTGCCCGAGTATCGTG |  |  |
| *aac(6’)-Ib-cr* | F: CTCGAATGCCTGGCGTGTTT | 482 | 24 |
|  | R: TTGCGATGCTCTATGAGTGGCTA |  |  |

*Ref, references

Supplementary Table S3. Comparison of antibiotic resistance genes (ARG) in the gut between the people with HARG and LARG

Q, quartile; MLS, macrolide-lincosamide-streptogramins; HARG, high antibiotic resistance gene; MARG, middle antibiotic resistance gene; LARG, low antibiotic resistance gene

Supplementary Table S4. Comparison of diet habit between people with HARG, MARG and LARG in the gut

HARG, high antibiotic resistance gene; MARG, middle antibiotic resistance gene; LARG, low antibiotic resistance gene
